# West Chicago Medical Society

**Published:** 1879-10

**Authors:** W. T. Belfield

**Affiliations:** Secretary


### ARTICLE XI.

WEST CHICAGO MEDICAL SOCIETY. Reported by W. T. BELFIELD, Secretary.

A special meeting of the West Chicago Medical Society was held Monday evening, August 11th, to discuss the pathology and treatment of the intestinal diseases of children. Dr. Harcourt was called to the chair. There were twenty-three ladies and gentlemen present.

DR. H. WEBSTER JONES enumerated among the causes of summer diarrhœa the effect of continuous heat upon the nervous centers; errors in diet; errors in clothing (from excess as well as deficiency in attire). He also discussed milk and the various substitutes for it, and the remedies needed in conjunction with proper diet. The doctor advised the allowance of plenty of water.

DR. EARLE recognized three diseases of this class (excluding dysentery): 1st. Simple diarrhœa; 2d. Entero-colitis; 3d. Cholera Infantum—the latter being, in his opinion, a very rare disease. He laid great stress on the regulation of the diet in both prophylaxis and treatment of these diseases. He thought the necessity for medicines in these cases to be less frequent than is commonly supposed.

DR. FOSTER referred to the fact that a large percentage of his cases began during the hot weather between June 27 and July 4. Dr. Bridge thought that a child often nursed simply to quench thirst, and that in summer, water should be usually offered before nursing was permitted. Dr. Lee referred to a case of severe infantile dysentery in which the greatest benefit had been derived from iodoform suppositories. Remarks were made also by Drs. Seeley and Clarke.

This discussion was continued at an adjourned meeting held August 18th. Dr. E. Ingals remarked that the heat, in order to be productive of bowel complaints, must be continuous for several days; that the nomenclature of these diseases should not be based upon the pathological conditions present, for these are not understood. He thought that they were seldom inflammatory; and that care should be taken that diet be not neglected for physic.

The doctor usually treated diarrhœa of the lower bowel by a cathartic followed by opiates. Farinaceous foods and cow's milk are desirable. He did not despair simply because a child is bottle-fed, for he had seen children thrive admirably when thus nourished. Nor did he think that a cow was necessarily unhealthy because inactive.

DR. LYMAN thought that the varying success of remedies in these diseases might be explained by a failure to distinguish stages and varieties of the difficulty. He briefly reviewed the minute anatomy of the intestine, dwelling especially on the connection of its nervous apparatus with the larger sympathetic ganglia. The doctor regards summer complaint as a succession of three stages: (1) Irritative—resulting often from nervous irritation, either external, such as a chill, or internal, *e.g.*, green apples in the alimentary canal. Equilibrium can be often restored by a bath and opium. Later, this stage merges into the (2) Inflammatory, wherein purgatives are most successful, especially mercurials; with these can be oftentimes profitably associated alkalies, to counteract acidity. The third stage is that of atony wherein tonics are demanded.

DR. LYMAN regards cholera infantum as a disease of depression, not of inflammation; that it is due, in part at least, to foul and malarious gases; that its frequency decreases as sewerage is perfected.

DR. EARLE spoke of the similarily between the symptomatology of epidemic cholera and cholera infantum. He took exception to Dr. Jones' recommendation that a sick child should have an abundance of water. Dr. Earle often forbids water or even the breast for 12 or 24 hours. Koumiss sometimes gives excellent results.

DR. GRAHAM narrated his experience with koumiss in one case—a four-months old child—who, after six weeks steady decline, showed marked and immediate improvement when put upon koumiss.

---

A SOAP FOR THE PHYSICIAN'S OFFICE.—MR. E. H. SARGENT, a well-known druggist of this city, has made a thymoline soap, designed especially for the antiseptic cleansing of physicians' instruments. The soap contains a sufficient quantity of the disinfectant, and the odor of the thymoline is by no means unpleasant. In this day, when so much is said respecting the transmission of disease by surgical instruments, it will be a satisfaction to make use of an agent in cleansing them, which will at least assist in their disinfection.

